# FES-UPP: A Flexible Functional Electrical Stimulation System to Support Upper Limb Functional Activity Practice

**DOI:** 10.3389/fnins.2018.00449

**Published:** 2018-07-05

**Authors:** Mingxu Sun, Christine Smith, David Howard, Laurence Kenney, Helen Luckie, Karen Waring, Paul Taylor, Earl Merson, Stacey Finn

**Affiliations:** ^1^Centre for Health Sciences Research, University of Salford, Salford, United Kingdom; ^2^Department of Allied Health Professions, Sheffield Hallam University, Sheffield, United Kingdom; ^3^School of Computing, Science and Engineering, University of Salford, Salford, United Kingdom; ^4^The National Clinical FES Centre, Salisbury District Hospital, Salisbury, United Kingdom

**Keywords:** functional electrical stimulation, upper limb, stroke, rehabilitation, finite state machine control

## Abstract

There is good evidence supporting highly intensive, repetitive, activity-focused, voluntary-initiated practice as a key to driving recovery of upper limb function following stroke. Functional electrical stimulation (FES) offers a potential mechanism to efficiently deliver this type of therapy, but current commercial devices are too inflexible and/or insufficiently automated, in some cases requiring engineering support. In this paper, we report a new, flexible upper limb FES system, FES-UPP, which addresses the issues above. The FES-UPP system consists of a 5-channel stimulator running a flexible FES finite state machine (FSM) controller, the associated setup software that guides therapists through the setup of FSM controllers via five setup stages, and finally the Session Manager used to guide the patient in repeated attempts at the activities(s) and provide feedback on their performance. The FSM controller represents a functional activity as a sequence of movement phases. The output for each phase implements the stimulations to one or more muscles. Progression between movement phases is governed by user-defined rules. As part of a clinical investigation of the system, nine therapists used the FES-UPP system to set up FES-supported activities with twenty two patient participants with impaired upper-limbs. Therapists with little or no FES experience and without any programming skills could use the system in their usual clinical settings, without engineering support. Different functional activities, tailored to suit the upper limb impairment levels of each participant were used, in up to 8 sessions of FES-supported therapy per participant. The efficiency of delivery of the therapy using FES-UPP was promising when compared with published data on traditional face-face therapy. The FES-UPP system described in this paper has been shown to allow therapists with little or no FES experience and without any programming skills to set up state-machine FES controllers bespoke to the patient’s impairment patterns and activity requirements, without engineering support. The clinical results demonstrated that the system can be used to efficiently deliver high intensity, activity-focused therapy. Nevertheless, further work to reduce setup time is still required.

## Introduction

In the United Kingdom there are more than 100,000 new stroke cases each year and approximately 1.2 million people living with the consequences of stroke (Stroke Association, 2017). In the United Kingdom, during their entire in-patient stay, a typical patient will receive around 5 h of physiotherapy (McHugh and Swain, 2014), with much of that time focused on the rehabilitation of posture, balance and walking (Wit et al., 2005). The consequences of this are that patients do not receive anything approaching the intensity of upper limb therapy that research suggests is needed to drive functional recovery (Clarke et al., 2015). Possibly as a result, long term recovery of the upper limb remains very poor. Almost three quarters of stroke survivors are left with upper limb motor problems (Lawrence et al., 2001), which seriously impact on their quality of life.

There is strong evidence supporting intensive (Lohse et al., 2014), repetitive, activity-focused (Winstein et al., 2004; Alon et al., 2007; Langhorne et al., 2009), voluntary-initiated (Peckham and Knutson, 2005; Knutson et al., 2009) practice for upper limb functional recovery. However, to enable such an approach, without significantly increasing the number of therapists, we need to look to rehabilitation technologies.

A number of rehabilitation technologies have been developed to encourage the recovery of upper limb motor function after stroke, including robotic devices, virtual reality and functional electrical stimulation (FES) systems (Howlett et al., 2015). Studies have shown positive results for FES in the rehabilitation of reaching and grasping function (Thrasher et al., 2008; Knutson et al., 2009), elbow extension (Thrasher et al., 2008; Hughes et al., 2010), shoulder motion (Hara et al., 2009), and stabilization of wrist joints (Malešević et al., 2012). In addition, FES offers the potential to increase therapy dose at a reasonable cost (Kitago and Krakauer, 2013), in a way that does not need the dedicated attention of a therapist.

Current upper limb FES systems can be categorized according to the methods of control over stimulation. The first group of systems use a push button operated by the patient’s unaffected hand, and/or are pre-programmed to repeat a fixed sequence of timed stimulations (Mann et al., 2005). Commercial systems of this type, which tend to be used largely for passive exercising, include Odstock Medical’s Microstim 2 and 4 Channel Stimulator Kit, and the Bioness H200. The Odstock 2 and 4 channel stimulators offer flexibility over which muscles are stimulated; the H200 (Snoek et al., 2000) offers 5 channels of stimulation, but is limited to stimulation of hand and wrist. Previous studies have suggested that cyclical stimulation is less clinically effective than voluntary triggered stimulation (de Kroon et al., 2005), although debate on this issue continues (Wilson et al., 2016). A recent report identified that the carryover, or therapeutic effect, in drop foot patients was only observed in patients who showed brain activation patterns consistent with movement planning (Gandolla et al., 2016). This supports Rushton’s hypothesis (Rushton, 2003) which proposed that when the F wave resulting from stimulation coincides with voluntary intention to move, connectivity between the intact upper motor and lower motor neurons is strengthened at the spinal cord level. These studies suggest that stimulation delivered without the active involvement of the patient may not be the most effective approach.

The second group of systems attempt to ensure that stimulation coincides with voluntary intention to move; thus increasing the likelihood of effective motor relearning. Examples of systems which use voluntary initiated neural signals to control FES include the EMG-based MeCFES (Thorsen et al., 2001) and STIWELL med4 (Rakos et al., 2007) systems and a small number of demonstrator projects which use brain-computer interface approaches (Müller-Putz et al., 2005; Ajiboye et al., 2017). However, reliable surface EMG signal(s) from appropriate muscles are frequently either difficult to measure or absent in people with paretic upper limbs (Bolton et al., 2004; Gazzoni, 2010), making EMG-controlled FES difficult to use with certain patients. Additionally, the voluntary effort in producing an EMG signal can increase spasticity, opposing the movement that is intended. Although systems using brain-implanted electrodes have been reported, most of the current EEG controlled systems use non-invasive electrodes, which provide limited information transfer rate, require patients to complete a significant amount of training prior to first use (Scherberger, 2009; Bouton et al., 2016), and need frequent re-calibration (Ajiboye et al., 2017).

Motion-controlled FES systems offer an attractive alternative (Mann et al., 2011; Sun et al., 2016a,b). An example of a motion controlled system is the Bionic Glove (Prochazka et al., 1997) which uses data from a wrist position sensor to control stimulation of hand and wrist muscles in C6/7 spinal cord injury (SCI) patients. More recently, the Southampton group have reported on a system based on iterative learning control (Meadmore et al., 2014) in which stimulation is applied to the triceps, anterior deltoid and wrist/finger extensors muscles to support specified reaching activities. Stimulation levels are adjusted cycle-to-cycle based on kinematic data collected from previous attempts in such a way that the patient is always challenged. These motion controlled FES systems have the potential to deliver appropriately timed neural inputs to promote re-learning and hence recovery (Rushton, 2003; Sheffler and Chae, 2007) and recent studies have reported positive results (Knutson et al., 2012; Meadmore et al., 2014), including sustained improvements in function (Persch et al., 2012), and improvements even in patients with severe hand arm paralysis (Popovic et al., 2005; Thrasher et al., 2008). However, these systems are generally inflexible in terms of the number and location of muscles to be stimulated (Snoek et al., 2000; Alon and McBride, 2003; Mann et al., 2011) and/or require engineering support to accommodate a wide range of upper limb activities (Tresadern et al., 2008). Relatively little attention has been paid to the development of easy to use, flexible systems able to support a range of patients in practicing varied, yet challenging functional activities (Rakos et al., 2007; Tresadern et al., 2008). In particular, if such systems are to be widely adopted, they must be sufficiently user-friendly to remove the need for routine engineering support.

In this paper, we report on a new, flexible upper limb FES system, FES-UPP, which address the issues discussed above. Below we report on the design of the upper limb FES controller and the setup software. Finally, we show data from a clinical investigation study of the system carried out without on-site engineering support to illustrate the potential for the system to be used in the delivery of intensive FES-supported practice.

## Materials and Methods

The aim is to enable therapists to quickly and easily configure the FES-UPP system to deliver FES-support during patient-specific upper-limb functional activities. In this section, after providing an overview of the system, we describe the design of the FES-UPP flexible finite state machine (FSM) controller, the associated setup software that guides therapists through the set-up of FSM controllers, and finally the Session Manager used to guide the patient and provide feedback on their performance.

### System Overview

**FIGURE 1:**
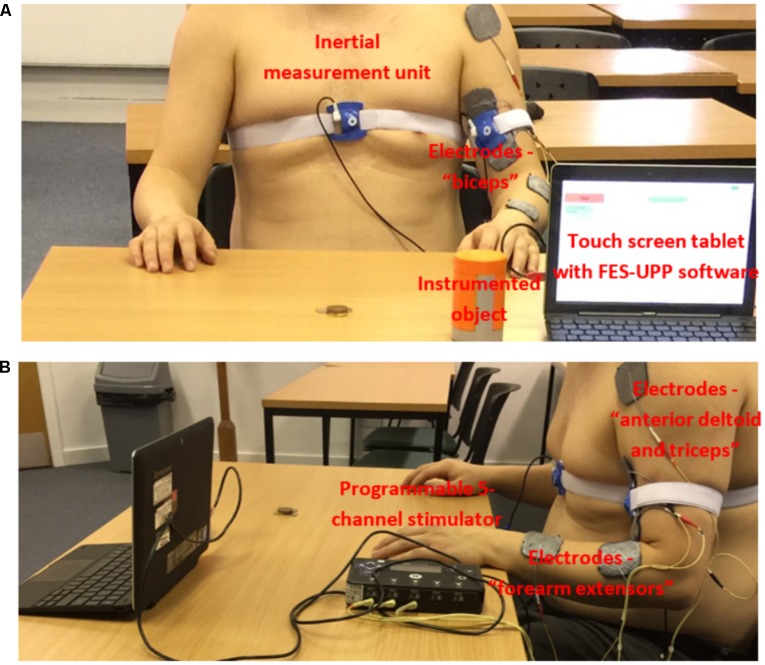
Example set-up of the FES-UPP system for the “Sweeping coins” activity. **(A)** Anterior view; and **(B)** Lateral view (informed consent was obtained from all participants).

Referring to **Figure 1**, the system consists of: a programmable 5 channel stimulator; a touch screen tablet with software for setting up and managing therapy sessions; an instrumented object; and up to four inertial measurement units, each containing a 3 axis accelerometer and a 3 axis rate gyroscope.

The battery-powered stimulator (measuring 30 × 160 × 96 mm) contains two 32-bit microprocessors (for control and data processing) and 5 stimulation channels, each with its own microcontroller. The outputs are individually charge balanced and safety isolated (EN 60601-1 type BF). The stimulator has wired interfaces supplying power to and receiving data from external sensors and the wireless Zigbee interface supports bi-directional data for sensors, remote control and system monitoring. The stimulator also has an isolated USB interface, built-in flash memory and an SD-card slot.

### Design of the Flexible Finite State Machine Controller

The flexible FSM controller design has been reported in detail by Sun et al. (2016a). In summary, the controller represents a functional activity as a sequence of movement phases. Each phase implements the ramping of muscle stimulation(s) toward their respective targets and then holds them at those targets. Progression between movement phases implements therapist-defined rules, which may be based on data from body-worn inertial sensors (**Figure 1**), an instrumented object, a button, or clock time. The instrumented object detects when a patient grasps, releases, or replaces the object onto a surface.

The inertial sensors provide Euler angles. The Euler angles rotation sequence is body segment-specific, in order to minimize the chance of gimbal lock. Body segment angles from the sensor(s) are streamed into the FSM controller in real time during the performance of a functional activity. Apart from angles, transition rules can also use a button press, timeout and instrumented object functions. To extend the flexibility of the system, logical operators (AND or OR) can be used to combine a maximum of two Boolean conditions (A and B) to create a transition rule.

**FIGURE 2:**
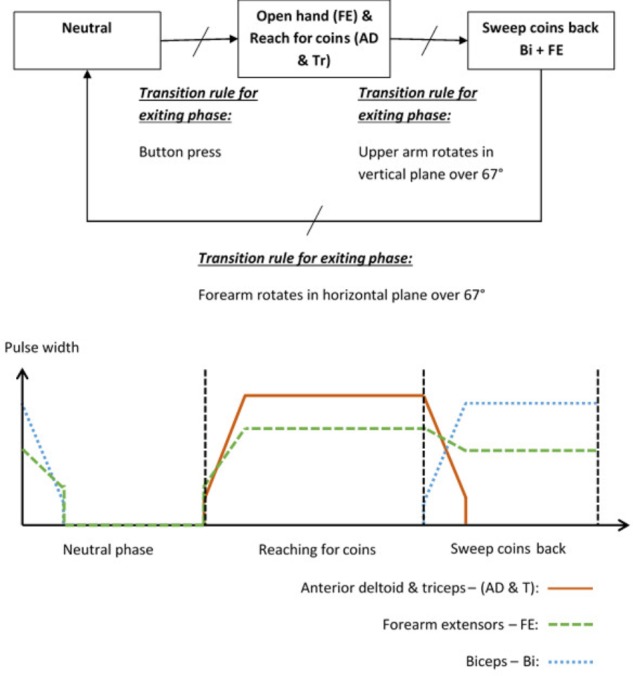
Example FSM and stimulation profiles for the “sweeping coins” activity.

To illustrate the way in which a patient and activity specific FSM controller can be implemented, the “Sweeping coins” example is described. The patient activity is to reach forward to a pile of coins on the table in front of them, then sweep the coins back toward them. Referring to **Figure 2**, this FSM has a neutral phase and two movement phases: “*Reach for coins*” *and* “*Sweep coins back*.” Each movement phase output function contains a set of muscles to be stimulated and their associated stimulation parameters. The sequence of movement begins after a button press on the tablet screen. In this example, phase 1 is used to open a hand and reach for coins. Stimulation is applied to the forearm extensors (FE) to open the hand and extend the wrist, and anterior deltoid and triceps muscles (AD and Tr) to flex the shoulder and extend the elbow. In phase 2, anterior deltoid and triceps stimulation is discontinued and the biceps (Bi) are stimulated to flex the elbow, allowing the coins to be swept back toward the body. The FE continue to be stimulated, but at a lower level, to maintain the hand in an open position with slight finger flexion. Transitions between phases are instantaneous events that occur on satisfaction of the *transition condition rules*. In this example, the transition between phase 1 (open hand and reach for coins) and phase 2 (sweep coins back) will be triggered by the angle of the upper arm increasing by an angle chosen by the therapist, for example 67°, since entering that phase. Alternatively, if the patient cannot achieve the required voluntary shoulder flexion, the therapist/patient can force this phase transition by repeating the button press. Each of the parameters listed above are defined by the therapist, depending on the chosen activity and the patient’s pattern of impairment.

#### Angle Trigger Algorithm

Stroke impaired patients tend to exhibit a higher degree of trial-to-trial kinematic variability when performing functional activities than healthy controls (Thies et al., 2009). It is therefore unlikely that a patient would follow exactly the same trajectory, or return their hand to exactly the same starting position after each attempt. This may lead to problems with consistently transitioning between phases using a transition rule based only on an angle change exceeding a given threshold value. Therefore, we have implemented a dual angle trigger algorithm in which a transition is triggered either when the change in angle since entering the phase exceeds a therapist-defined primary threshold, or exceeds a secondary (lower) threshold and is maintained above this threshold for at least 2 s. The secondary threshold is defined as 80% of the primary threshold.

In addition to the dual angle trigger algorithm outlined above, the therapist/patient can in all cases press a button to force a phase transition if the patient cannot achieve the required voluntary shoulder flexion.

### Design of the Setup Software for FSM Controllers

#### Breaking the Setup Process Into Logical Stages

**FIGURE 3:**
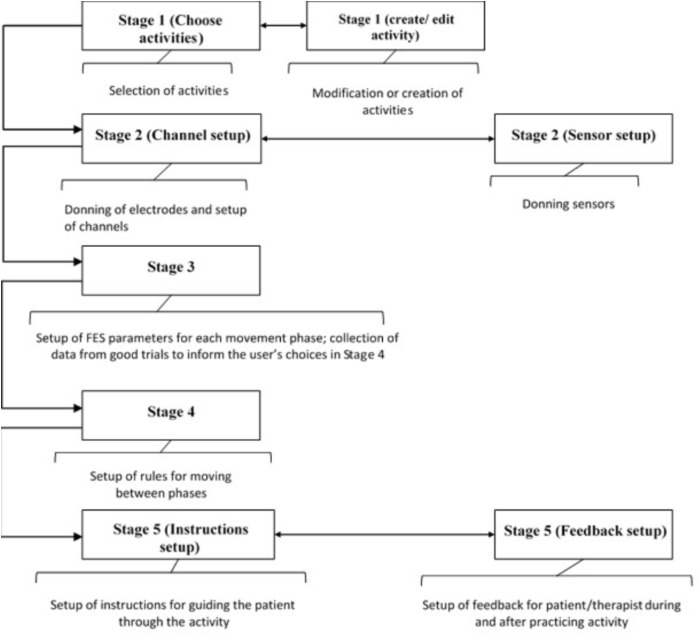
Overview of the five setup stages.

Tablet based setup software has been developed that guides therapists through the process of setting up bespoke FES controllers. The concept is to break down the setup of a FSM controller for a particular upper-limb activity into the following five logical stages shown in **Figure 3**:

(1) Selection, modification and/or creation of activities
(2) Donning of electrodes and sensors and set up of channels
(3) Set up of stimulation parameters for each movement phase
(4) Set up of transition rules
(5) Set up of patient instructions and biofeedback

Although the five stages follow a logical sequence, at any point, the therapist can move to any of the stages so long as the necessary prerequisites have been set up.

After going through these five setup stages, the therapist can leave this part of the software and enter the Session Manager, which allows the patient to practice the functional activity(s) and provides feedback to the therapist and patient on their performance, both during and after practice. The setup software stages are described in more detail in the following sections.

#### Stage 1 – Create, Modify and Select Activities

Prior to Stage 1, the therapist can create, modify and then select a patient record. Stage 1 then guides the therapist through the process of creating, modifying, and then selecting functional activities for a particular patient.

**FIGURE 4:**
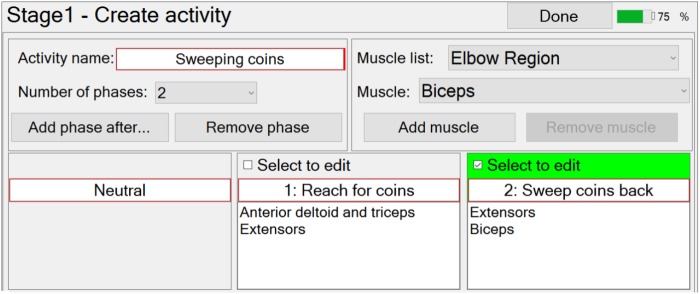
“Stage 1 Create/Edit activity” window for the example activity “Sweeping coins.”

At this stage in the setup process, the therapist can create a new functional activity by specifying the number and order of phases, each of which (apart from neutral) is associated with the muscles that are going to be stimulated in that phase (**Figure 4**). The number of phases can be edited via a drop-down menu, and the order can be edited using the “Add phase after…”, or “Remove phase” buttons. The required muscles can be selected from a drop-down menu and then associated with a selected phase by pressing the “Add muscle” button. Similarly, the therapist may remove muscles from a phase with the “Remove muscle” button. The therapist is required to enter a unique activity name and phase names, via text boxes.

Modification of an activity is carried out in a very similar way to creating a new activity.

FES-UPP also provides libraries of standard FES supported hand-arm activities so that therapists don’t have to start from scratch each time. Importing a functional activity is implemented via a pop-up window which shows the standard hand-arm activity libraries.

When the therapist is satisfied that the set of activities have been fully defined, the therapist can then select which one(s) to use in the current therapy session, via the “Use (selected activities) today” button (screen shot not shown).

#### Stage 2 – Don Electrodes and Sensors, and Initial Channel Setup

**FIGURE 5:**
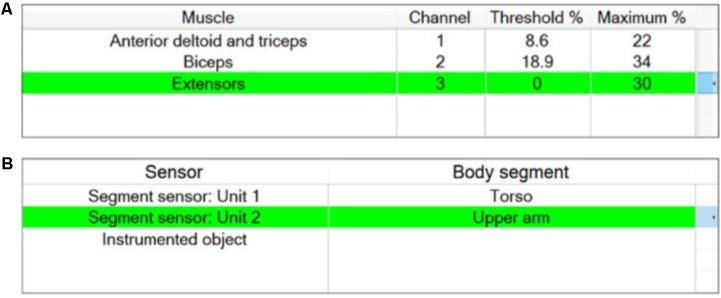
“Stage 2 – Setup of stimulator channels and sensors” window for the example activity “Sweeping coins.” **(A)** Channels assignment including setting maximum and threshold stimulation values; **(B)** Sensor assignment.

Stage 2 of the setup process involves associating stimulation channels with the muscles chosen in Stage 1, testing the placement of each electrode, setting maximum and motor threshold for each channel, and deciding on the set of sensors required. The therapist is presented with two windows, one window corresponding to the channel setup (**Figure 5A**), the other corresponding to the sensor setup (**Figure 5B**).

##### Donning and initial setup of stimulator channels

A maximum of 5 stimulation channels can be assigned. Unless otherwise specified by the therapist using the channel assignment buttons, the stimulation channels are automatically assigned to muscles in a proximal to distal order (**Figure 5A**). The stimulation parameters defined for each muscle are maximum comfortable and motor threshold (the minimum stimulation level to create a just detectable motor response). These numbers are used to determine the operating range within which the therapist can specify phase-specific target stimulation values.

The maximum comfortable stimulation levels for each muscle are found by ramping up pulse amplitude via an arrow key. During this process pulse width is fixed at 180 μs and the absolute maximum value that the stimulator can deliver is limited to 120 mA. The motor threshold is then found by increasing the pulse width until a detectable muscle response is produced (at the previously determined maximum comfortable pulse amplitude). The maximum comfortable stimulation and motor threshold are displayed as percentages of the total charge/pulse the stimulator is capable of producing, as shown in **Figure 5A**.

##### Donning and initial setup of sensors

In a process similar to the setup of stimulator channels, the therapist assigns the motion sensor units to different body segments. The set of available motion sensors is automatically provided by the system in a plug-and-play manner, as shown in the left column in **Figure 5B**, and the therapist associates sensors with body segments using assignment buttons. The sensor setup window also displays a plot of the sensor unit data in real time to guide the therapist during initial setup and test if a sensor is working properly. There is no additional information required from the therapist to set up the instrumented object and if it present, will be available for use in setup Stage 4.

#### Stage 3 – Setting Up Stimulation Parameters for Each Phase and Capturing Manual Transitions Data

**FIGURE 6:**
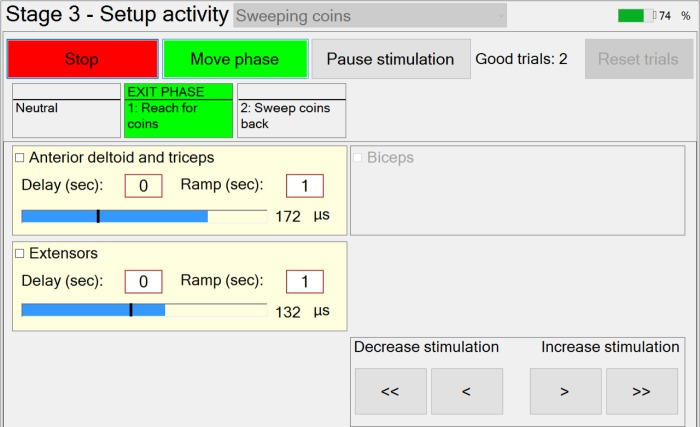
“Stage 3” window – adjusting stimulation profiles for example activity “Sweeping coins.”

**FIGURE 7:**
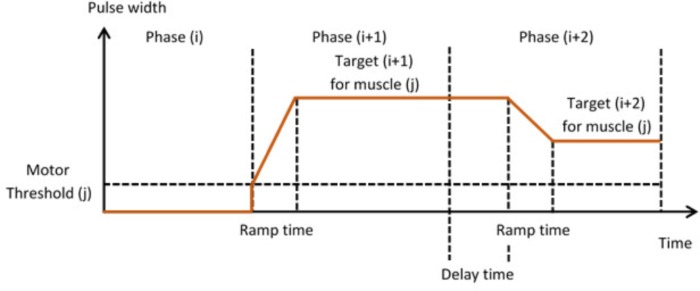
Stimulation profiles during consecutive movement phases, each consisting of a stimulation target, ramp time, and delay time.

In Stage 3, the therapist is guided through the setting up of suitable stimulation profiles (**Figure 6**) for each muscle in each phase. A stimulation profile consists of delay, ramp time and target value for each muscle in each phase. There are no muscles associated with the neutral phase. Referring to **Figure 6**, in each subsequent phase and for each active muscle, the therapist manually adjusts pulse width to find a suitable stimulation target that achieves the required movement in that phase. In addition, the delay and ramp time for each muscle in each phase are also defined by entering values in their edit boxes. Ramp times are designed to avoid sudden jumps in stimulation, and are the periods over which stimulation ramps from its previous target to its new target (**Figure 7**). Delays are the periods before stimulation ramping begins (**Figure 7**) and may be used to assist with coordination between stimulation channels. If stimulation becomes uncomfortable, it can be stopped at any time by pressing the “Pause” button. Pressing the button a second time restarts stimulation by ramping back to the level prior to pausing.

If multiple activities are available, the therapist can set up the stimulation profiles for each activity in turn, by selecting the relevant activity from the drop-down menu. While setting up the stimulation profiles, movement between successive phases is achieved via a button press. The transition rules allowing for automation of phase transitions are defined in setup Stage 4.

Once the stimulation profiles are acceptable, patients are required to perform the activity a number of times. After each attempt at the activity has been completed, the therapist will decide whether or not the activity was achieved successfully (a “good” attempt). For the “good” attempts, *time-spent-in-phase* and, for each instrumented body segment, *change-in-angle-since-entering-phase* are recorded for use in Stage 4 (i.e., for setting up phase transition rules). Immediately prior to leaving this stage, the captured transition data from the set of “good” attempts are averaged and passed as “suggested values” to Stage 4. In situations where gimbal lock may be an issue, the affected Euler angle is not passed on and that Euler angle cannot be used for triggering the transition.

#### Stage 4 – Setting Up Transition Condition Rules

Having set up satisfactory stimulation profiles for each muscle in each phase, the next stage of setup involves defining the transition rules for progressing from one phase to the next. A transition between two successive phases can be triggered by a button press, a timeout, a change in body segment angle since entering the phase, instrumented object functions, or a logical combination of two of these events. Transition rules can therefore take one of the following three forms: A; (A OR B); (A AND B).

The therapist may choose from one of the body segments to which a sensor has been assigned previously, and hence, in this example, ‘Hand’ and ‘Forearm’ are not available (**Figure 5B**).

When a body segment is selected, another “Option” drop-down menu is used to select the change in one of up to 3 possible angles. An angle threshold textbox offers a suggested value which is the average of the “good” attempts captured in Stage 3 (e.g., Upper arm rotates in vertical plane over 67°). The therapist can either accept or change the suggested angle threshold using the edit box.

If a ‘timeout’ has been selected, a textbox offers a suggested value. As above, the therapist can either accept or change this value. If an ‘Instrumented object’ has been selected, the therapist can select “Grasp object,” “Release object” and “Replace object,” because the instrumented object incorporates grip sensors and a switch on its base. If a ‘button’ press has been selected, no other information is required.

#### Stage 5 – Setting Up Activity Instructions and Feedback

Stage 5 guides the therapist through the setup of patient and activity-specific instructions and feedback on performance during practice of the activities. Activity instructions are text displayed on the tablet screen, which specify the particular goal in each of the phases (e.g., “reach for coins”). For each phase, the therapist may select from pre-defined patient instructions, via a drop-down menu, or input their own text. For the “Sweeping coins” example activity (**Figure 2**), a simple instruction window (screen shot not shown) can be used to set up the instructions “Reach to target with a long arm” and “sweep the coins back toward you,” for phases 1 and 2 respectively. However, the therapist can also choose not to provide any instructions if the instruction display would distract the patient from focusing on practicing the activity.

Three types of feedback are available. Firstly, feedback can be provided on the patient’s progress toward achieving the required movement (or time in phase) to trigger the transition to the next phase. This is in the form of a continuous visual display immediately beneath the instruction text, and/or a repeated sound, whose frequency depends on the magnitude of movement.

**FIGURE 8:**
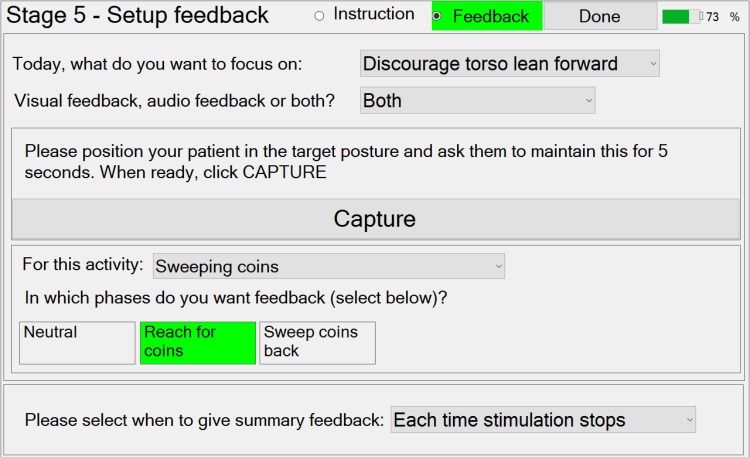
“Stage 5 – Setup feedback” window for the example activity “Sweeping coins.”

Secondly, trunk lean feedback can be provided, which is independent of the required movement. Making patients aware of their trunk movement is a commonly used technique in physiotherapy, either to discourage using trunk lean during a reach forward movement, or to encourage trunk lean during a sit-to-stand movement. If the therapist decides that trunk lean feedback would be useful in a phase, then they can choose to provide visual feedback (a moving arrow) and/or audio feedback (beeps). Referring to **Figure 8**, to setup trunk lean feedback, the system must first be provided with information on the target trunk lean (zero if lean is to be discouraged) using the “Capture” button.

Thirdly, the therapist can set up the summary feedback given at the end of each attempt, via a drop-down menu.

### Design of the Automatic Therapy Session Manager

Once the five setup stages described above have been completed, the Session Manager is used during a therapy session to guide the patient while they are practicing functional activities. The Session Manager appearance will be dependent on the nature of the activity/activities that have been set up, but is always divided into three parts: a control panel, an instruction panel and a feedback panel.

The control panel consists of a “Start/Stop” stimulation button and a “Move phase” button. Once the “Start stimulation” button has been pressed, the color of this button changes from green to red and the text changes from “Start” to “Stop.” Subsequently, pressing this button will immediately stop stimulation to all muscles. Moving phase can be achieved at any time by pressing the “Move phase” button.

**FIGURE 9:**
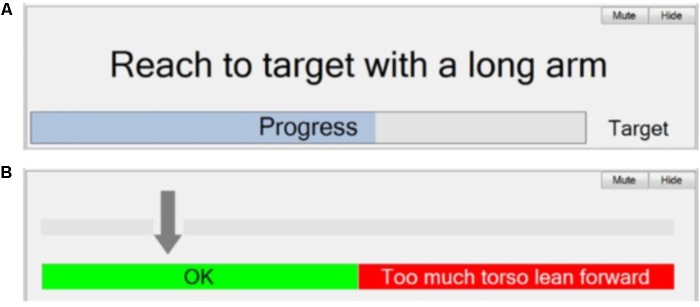
Automatic therapy Session Manager. **(A)** Instruction panel – Text instruction and visual feedback to the patient/therapist; and **(B)** Feedback panel – visual feedback of trunk lean to the patient/therapist.

The instruction panel consists of a display to provide text instructions guiding the patient on the movement required in the current phase. It also includes feedback on the patient’s progress toward achieving the required movement (or time in phase) to trigger the transition to the next phase. This is in the form of a continuous visual display immediately beneath the instruction text, and/or a repeated sound, whose frequency depends on the magnitude of movement. An example for the “Sweeping coins” activity is shown in **Figure 9A**. The window shows the instructions set up in Stage 5 (“Reach to target with a long arm”). The visual feedback bar provides real-time tracking of progress toward the target upper-arm angle (trigger threshold).

**Figure 9B** shows an example of feedback of torso angle. The intention here is discourage the patient from leaning forward while reaching forward with their arm. The arrow indicates the angle of the torso from vertical. An audio warning is given if the arrow moves into the red.

### FES-UPP Implementation

The Flexible FSM controller runs on an Odstock 5 Channel Stimulator (Merson et al., 2017). The FES-UPP software was coded using C# windows forms in Microsoft Visual Studio 2010 under the Windows 7 Enterprise platform. Microsoft Visual Studio 2010 was used to publish the C# windows forms application, creating a stand-alone executable file which can be run on any windows tablet PC. The FES-UPP software communicates with the Odstock 5 Channel Stimulator via a micro USB.

#### Data Logging

The software on the tablet also automatically creates a set of logged data files for each therapy session. The logged data files include setup information for each activity (i.e., Patient ID, number of movement phases, muscles stimulated during each phase, transition rules, stimulation parameters) as well as information corresponding to each activity repetition (i.e., activity name, repetition number, time spent in each movement phase, transition rules used in each phase transition, whether or not the repetition was successful). Key interaction events between the therapist and FES-UPP software are also logged (i.e., the therapist ID, FES-UPP log on/off, entering setup stages and Session Manager etc.).

### Use of the System in a Clinical Investigation

To illustrate the use of the system, we report data for 22 patients and 11 therapists from a recently completed clinical investigation of the system (REC ref 16/YH/0258)^1^. The clinical investigation took place at three different clinical sites in the United Kingdom. The primary aim of the study was to demonstrate that use of the FES-UPP enables participants to perform a wider range of functional activities, and/or perform the same activities in an improved way. A secondary aim was to evaluate the usability of the system. Here we present relevant data from this clinical investigation to illustrate the usability of the system.

Participants with stroke were treated by therapists using the system on up to 8 therapy sessions, spread over up to 6 weeks. Prior to starting the study, all therapists attended a 2-day training session in order to familiarize themselves with the FES-UPP system and the study protocol. A clinical manual and an on-line training resource for the system were also provided to the therapists for use during the study. The patient and therapist participants are described in **Table 1**.

**Table 1:** Participants.

Patient participants					

ID	Age range (years)	Hand dominance	Time since CVA	Affected Side	FM UL/66
X01	80–85	R	<1 week	R	8
X02	50–55	R	<1 week	R	12
X03	25–30	R	2 weeks	R	6
X04	60–65	R	2 weeks	L	49
X05	80–85	R	<1 week	L	4
X06	85–90	L	<1 week	L	10
X07	50–51	L	3 weeks	R	4
Y01	65–70	R	26 weeks	R	34
Y02	70–75	R	23 weeks	L	8
Y03	65–70	R	7 weeks	L	13
Y04	65–70	R	21 weeks	L	5
Z01	60–65	R	6 weeks	R	10
Z02	45–50	R	3 weeks	L	11
Z03	45–50	R	17 months	R	6
Z04	70–75	R	126 months	L	2
Z05	80–85	R	4 weeks	R	2
Z06	70–75	R	234 months	R	0
Z07	45–50	R	39 months	L	10
Z08	80–85	R	71 months	L	0
Z09	75–80	R	28 months	L	12
Z10	75–80	R	8 weeks	R	12
Z11	60–65	R	9 weeks	L	10

**Therapist participants**					

**ID**	**Role**		**Clinical experience (year)**	**Previous FES experience**	

XOT1	Occupational therapist		15	No	
XPT1	Physiotherapist		6	Yes	
XRA1	Rehabilitation Assistant		1	No	
YOT1	Occupational therapist		7	No	
YPT1	Physiotherapist		16	No	
YPI1	Stroke Specialist Nurse		12	No	
YRA1	Rehabilitation Assistant		10	No	
YRA2	Rehabilitation Assistant		1	No	
ZPT1	Physiotherapist		21	Yes	
ZCE1	Clinical Engineer		5	Yes	
ZCE2	Clinical Engineer		30	Yes	


#### Participants

In this paper we report on two usability metrics, efficiency and completion rate.

We define efficiency as follows:

$$\text{Efficiency}=\frac{\text{the total practice time in a therapy session}}{\text{the total therapy time}}\times 100\%$$

We define completion rate as follows (Smith, 2015):

$$\text{Completion rate for a therapy session}=\frac{\text{number of successful repetitions of activity}\left(s\right)}{\text{the total number of attempts at the activity}\left(s\right)}\times 100$$

#### Example Setup

As an example, **Figure 1** shows the clinical setup for the “Sweeping coins” activity. Motion sensors were attached to the front of the chest and upper arm of the patient participant using adjustable sensor straps. In this case, the participant was asked to carry out the “Sweeping coins” activity assisted by electrical stimulation to the muscle groups “FEs,” “biceps” and “anterior deltoid and triceps,” as shown in **Figure 1**. The activity was imported from a standard hand-arm activity library by the therapist using the FES-UPP setup software.

## Results

**FIGURE 10:**
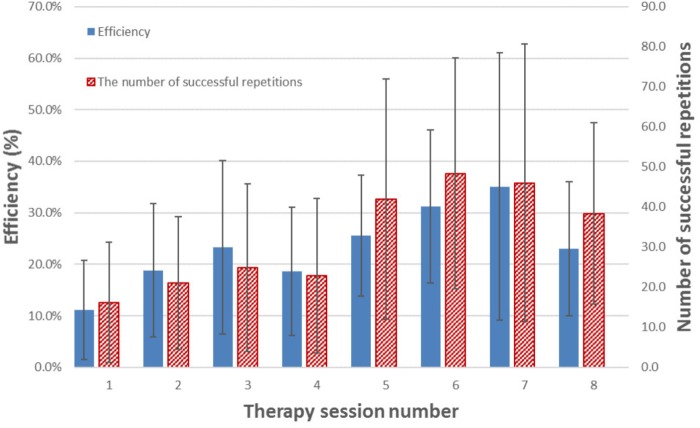
Mean efficiency and mean number of successful repetitions of activity(s) for each therapy session across all patient participants who managed to complete that session. The error bars indicate standard deviation over the patient participants who completed that session. Please note, not all participants are managed to complete all 8 sessions. **Table 2** illustrates the number of session completed for each participant.

Apart from YPT1 and YPI1, all therapist participants used the FES-UPP system to support functional activity practice with one or more patient participants on one or more occasions. For each participant and up to 8 sessions undertaken^2^, **Figure 10** shows the mean efficiency and mean number of activity repetitions for each therapy session across all 22 patient participants and. There is a clear increase in both efficiency and number of repetitions toward the end of the study across all participants.

**Table 2** illustrates the total number of therapy sessions completed, total repetition number for those completed therapy sessions and the completion rates across participants.

**Table 2:** The completion rate for each participant across varying number of therapy sessions.

Participant	X01	X02	X03	X04	X05	X06	X07	Y01	Y02	Y03	Y04
Session number	2	7	7	3	8	3	4	5	2	2	1
Total repetitions	18	256	215	18	108	38	81	36	13	7	1
Completion rate (%)	100	96.1	100	100	100	97.4	100	86.1	100	57.1	100

**Participant**	**Z01**	**Z02**	**Z03**	**Z04**	**Z05**	**Z06**	**Z07**	**Z08**	**Z09**	**Z10**	**Z11**

Session number	8	7	8	8	2	4	8	8	1	5	4
Total repetitions	172	260	382	469	130	104	275	327	17	103	48
Completion rate (%)	95.3	86.5	97.9	98.1	88.5	95.2	95.3	95.4	88.2	94.2	95.8


## Discussion

Intensive and repetitive activity training are believed to be two of the key ingredients driving recovery of the upper limb post stroke (French et al., 2009, 2010). This paper has introduced an advanced FES system for upper limb rehabilitation, FES-UPP, which allows therapists without any programming skills to quickly and easily set up controllers to deliver FES-support for patient-specific upper limb functional activities. **Figure 10** and **Table 2** illustrate the performance of the FES-UPP system in supporting early stage stroke patients to practice many repetitions of therapist-created, FES-assisted upper-limb activities.

In the clinical data from the clinical investigation presented here, 11 different therapists attended a 2-day training session and 9 of them used the FES-UPP system to set up FES-supported activities with 22 patient participants with severely impaired upper-limbs (**Table 1**). The therapist participants have varying clinical experience from 1 to 30 years. Four of them had previous FES experience. All the FES-supported activities used in this clinical investigation were either created from scratch, re-used from previous sessions, or modified from activities taken from a standard hand-arm activity library.

**Figure 10** illustrates a clear increasing trend in the efficiency of therapy delivery and number of repetitions of activity(s) over time. The increase in efficiency may be explained by a number of factors: firstly, as the therapists used the system more, they became quicker at setting it up; secondly, the most efficient delivery was carried out by the therapy assistant, who expressed a high degree of confidence in using the software and was able to re-use (with minor modifications) library activities which had been set up by a more clinically experienced physiotherapists or occupational therapist. Compared to creating an activity from scratch, setup time for a modification of an existing activity is somewhat lower. Indeed, setup time is an important, but neglected research area in the field of rehabilitation technologies (Smith et al., 2018). In the ideal case, a therapist would be able to retrieve settings from a previous session and go quickly to the Session Manager. However, further work is needed to understand how day-to-day variations in electrode placement and muscle response affect the stimulation settings.

It is worthwhile to compare the dose of therapy achieved in our study with the clinical reality of traditionally delivered physiotherapy (Hayward and Brauer, 2015). In a recent paper, Jong et al. (2017) studied 46 stroke patients with poor arm function from three rehabilitation centers in the Netherlands for 8 weeks, finding that physiotherapists and occupational therapists spent on average 4–7 min on arm-treatment per 30 min therapy session. Other papers (Lang et al., 2009; Kimberley, 2010) support this finding, reporting that in the inpatient setting the number of repetitions in a given session were on average 23 or less.

Apart from patient participant Y03, very high successful completion rates (greater than 86.1%) were achieved for all participants (**Table 2**). YO3 had the lowest completion rate of 57.1%, although this number should be treated with caution as the total number of repetitions was small (only 7 attempts for the activity(s), 4 of which were successful). Seventeen out of 22 participants achieved successful completion rates of over 90%. Part of the reason for this was that most therapists chose relatively simple activities in terms of the number of phases, number of stimulation channels, and complexity of the transition rules.

## Conclusion

The traditional approach to delivering upper limb therapy following stroke is extremely labor intensive. In practice, due to staffing limitations, patients receive very low ‘doses’ of therapy, which is likely contributing to the poor long term outcomes. These observations suggest that easy-to-use technology is urgently needed to improve the efficiency of therapy delivery, and thereby increase the ‘dose’ offered, particularly in the critical first few months following a stroke. In this paper, we have presented an FES system, consisting of a 5 channel programmable stimulator, tablet-based setup and feedback software and a range of sensors. The software guides the therapist through the setup of FSM controllers, bespoke to the particular activity and patient’s pattern of impairment. Transitions between states are governed by user-defined rules, which can be exploited to encourage voluntary effort on the part of the patient. The software also provides the patient and/or therapist with instruction and feedback on performance. We reported results from a study of the system being used without engineering support in the very early post-stroke period with 22 patients who had a range of arm impairments. The therapists with little or no FES experience and without any programming skills could use FES-UPP system to set up a range of functional activities. The results demonstrated that in most cases the system was used to deliver high intensity, activity-focused therapy. The efficiency with which the therapy was delivered was clearly better than seen in observational studies of face-face upper limb therapy. Nevertheless, further work to reduce setup time is still required.

## Data Availability Statement

The raw data supporting the conclusion of this manuscript will be made available by the authors, without undue reservation, to any qualified researcher.

## Ethics Statement

The study, within which the case study data was collected, was approved by the NHS (ref: 16/YH/0258) and University of Salford Research Ethics Committees (HSCR 16-39). The applicants also received a notice of no objection from the UK Medicines and Healthcare Regulatory Authority to the use of the FES-UPP system for the purposes of the clinical investigation study (C 1/2016/0034). The procedures employed in the study complied with ICH-GCP and The Declaration of Helsinki (2008). All participants signed an informed consent form prior to any study-related procedure.

## Author Contributions

MS, DH, LK, and CS contributed conception and design of the study. MS and DH contributed conception and design of the FES-UPP FSM controller and setup software. CS, PT, and LK designed the clinical trials. MS wrote the first draft of the manuscript and wrote the setup software code. CS, HL, and KW supervised and collected the therapist and patient study data. PT, SF, and EM designed the hardware 5 channel stimulator. EM implemented the hardware 5 channel stimulator. EM, MS, and DH designed the communication protocol between FES-UPP software and hardware stimulator. All authors contributed to manuscript revision, read, and approved the submitted version. MS and CS equally contributed to preparation of this article.

## Conflict of Interest Statement

The authors declare that the research was conducted in the absence of any commercial or financial relationships that could be construed as a potential conflict of interest.

## Footnotes

1 In this paper we use the term therapist to refer to clinically trained users of the system. In our study, the system was used by physiotherapists, occupational therapists, therapy assistants, clinical engineers, and a nurse.

2 In this paper we report data only on sessions where the therapist progressed through to the Session Manager stage of the software.
